# The Performance of a Multi-Stage Surface Flow Constructed Wetland for the Treatment of Aquaculture Wastewater and Changes in Epiphytic Biofilm Formation

**DOI:** 10.3390/microorganisms13030494

**Published:** 2025-02-22

**Authors:** Chuanxin Chao, Shen Gong, Yonghong Xie

**Affiliations:** Institute of Subtropical Agriculture, Chinese Academy of Sciences, Changsha 410125, China; chaochuanxin94@163.com (C.C.); GShikidowumi@163.com (S.G.)

**Keywords:** multi-stage SFCW, epiphytic biofilm, growth period change, water quality, removal efficiency

## Abstract

Constructed wetlands play a critical role in mitigating aquaculture wastewater pollution. However, the comprehensive treatment performance of aquatic plants and microorganisms under various water treatment processes remains insufficiently understood. Here, a multi-stage surface flow constructed wetland (SFCW) comprising four different aquatic plant species, along with aeration and biofiltration membrane technologies, was investigated to explore the combined effects of aquatic plants and epiphytic biofilms on wastewater removal efficiency across different vegetation periods and treatment processes. The results demonstrated that the total removal efficiency consistently exceeded 60% in both vegetation periods, effectively intercepting a range of pollutants present in aquaculture wastewater. Changes in the vegetation period influenced the performance of the SFCW, with the system’s ability to treat total nitrogen becoming more stable over time. The removal efficiency of the treatment pond planted with submerged plants was highest in July, while the pond planted with emergent plants showed an increased removal rate in November. The aeration pond played a significant role in enhancing dissolved oxygen levels, thereby improving phosphorus removal in July and nitrogen removal in November. Additionally, the α-diversity of epiphytic bacteria in the aeration and biofiltration ponds was significantly higher compared to other ponds. In terms of bacterial composition, the abundance of Firmicutes was notably higher in July, whereas Nitrospirota and Acidobacteriota exhibited a significant increase in November. Furthermore, the functional genes associated with sulfur metabolism, nitrogen fixation, and oxidative phosphorylation displayed significant temporal variations in the aeration pond, highlighting that both growth period changes and treatment processes influence the expression of functional genes within biofilms. Our findings suggest that the integration of water treatment processes in SFCWs enhances the synergistic effects between aquatic plants and microorganisms, helping to mitigate the adverse impacts of vegetation period changes and ensuring stable and efficient wastewater treatment performance.

## 1. Introduction

Aquaculture is a rapidly expanding global industry driven by the growing demand for aquatic products. This expansion has resulted in a substantial increase in aquaculture wastewater production, which presents significant environmental challenges (Kurniawan et al., 2021 [1]; Kashem et al., 2023 [2]). Aquaculture wastewater is distinct from other forms of wastewater, such as municipal or industrial effluent, due to its unique composition, which includes elevated levels of organic matter, nitrogen, phosphorus, and other nutrients derived from aquatic animals and feed (Vymazal, 2009 [3]; Vymazal, 2014 [4]). These characteristics pose specific environmental concerns. While aquaculture plays a vital role in food security and economic development, improper management of wastewater from these systems can lead to the severe degradation of surrounding water bodies, disrupt ecological balance, and threaten biodiversity (Cardoso et al., 2022 [5]; Kashem et al., 2023 [6]). Additionally, the inefficient use of water resources in aquaculture exacerbates these environmental issues, hindering the sustainable growth of the industry. Inland aquaculture systems, in particular, face more complex challenges compared to marine or coastal aquaculture, given their relatively closed environments, which can lead to more concentrated and difficult-to-manage wastewater characteristics (Edwards, 2015 [7]). Consequently, the development of effective and sustainable treatment models for inland aquaculture wastewater is crucial for promoting environmentally responsible practices and ensuring resource-efficient management within the industry.

A constructed wetland is a unique plant–bacteria–soil ecosystem built by imitating natural wetlands and comprises aquatic plants, bacteria, and artificial substrates, which play an important role in regulating material circulation and energy flow in wetlands (Sandoval et al., 2019 [8]; Wang et al., 2020 [9]). Combined with a variety of processes, constructed wetlands can provide diversified water purification functions suitable for treating high-load or large-flow wastewater (Gizinska-Gorna et al., 2020 [10]). In addition, the constructed wetland can be optimized according to specific water characteristics and treatment needs. The process combination and configuration of the constructed wetland can be adjusted to adapt to different environmental conditions and water quality characteristics, and the water purification effect can be improved (Ilyas and van Hullebusch, 2019 [11]). Therefore, studies on the applicability and effects of wetlands can provide an effective solution for the treatment of wastewater comprising complex pollutants and a scientific basis for the planning and construction of wetland systems.

Aquatic plants play various direct and indirect roles in constructed wetlands, with one of the most obvious being their direct absorption of nitrogen, phosphorus, and other nutrients (Gacia et al., 2019 [12]). In addition, the plant body provides sufficient space for bacteria to attach to and form a heterogeneous membrane with rheological properties on the surface of the plant (Lewandowski et al., 1995 [13]; Wolters et al., 2019 [14]). Epiphytic biofilms comprise diverse bacterial populations with varying environmental tolerances, possess distinct biological characteristics that serve as useful ecological indicators, and help consume nutrients in water and remove pollutants (Zhang et al., 2016 [15]; Zhu et al., 2021 [16]). As a result, they play a crucial role in pollutant transformation in aquatic ecosystems and maintaining ecological balance. Previous studies have revealed that plant-attached bacteria can actively respond and adapt to changes in different influent conditions, with corresponding changes in community structure and function (Engida et al., 2021 [17]; Zhang et al., 2018 [18]; Zhang et al., 2018 [19]). In constructed wetlands, differences in aquatic plants and water treatment processes can alter the heterogeneity of the water environment and indirectly affect bacterial communities (Chen et al., 2022 [20]; Fan et al., 2016 [21]; He et al., 2020 [22]; Xia et al., 2020 [23]). In addition, bacterial succession in epiphytic biofilms showed the existence of significant seasonal changes and different adaptation strategies in the biofilm communities during different seasons (Lyautey et al., 2005 [24]; Wang et al., 2022 [25]). Studies have shown that the community composition, functional traits, key enzymes, and key metabolic pathways of epiphytic biofilm bacteria change seasonally (Tian et al., 2023 [26]). Therefore, with the changes in aquatic plants, environmental factors, and treatment processes, the composition and function of epiphytic biofilms must be further explored.

In this study, we investigated the treatment process of aquaculture wastewater in July and November using a multi-stage surface flow constructed wetland (SFCW) system, monitored the treatment effects of different treatment ponds on aquaculture wastewater, and studied the biofilms attached to the surface of aquatic plants in six treatment ponds. High-throughput 16S rRNA amplification sequencing was used to analyze the bacterial community diversity, and the hypotheses were as follows: (1) the treatment effect of the SFCW on aquaculture wastewater is affected by the vegetation period; (2) there are differences in the removal efficiency of aquaculture wastewater in different treatment ponds; and (3) the diversity and structure of biofilm-attached bacteria will change with the variation of vegetation period.

## 2. Materials and Methods

### 2.1. Study Site and Sample Collection

**Figure 1:**
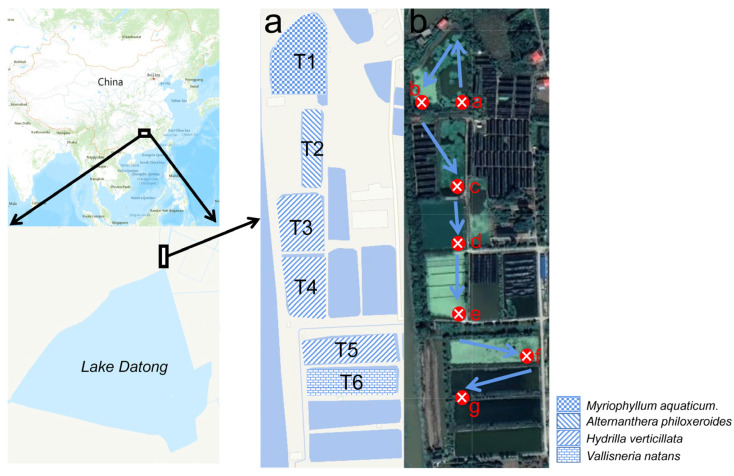
Location of sampling points of constructed wetland. The blue arrow shows the direction of the water flow; different capital letters (T1–T6) indicate the treatment pond (**a**); different lowercase letters (a–g) indicate the sampling point site (**b**).

The six-stage surface flow constructed wetland (SFCW) utilized in this study is located adjacent to a bullfrog (*Lithobates catesbeianu*) breeding facility in the northern part of Lake Datong, Yiyang City, Hunan Province, China (Figure 1). This SFCW was specifically designed to treat wastewater from bullfrog farming, ensuring its safe discharge into Lake Datong. The climate of this region is subtropical, with an annual precipitation of 1465 mm and an annual temperature of 17.6 °C. The SFCW comprises six stages: a primary sedimentation pond (T1), a secondary sedimentation pond (T2), an aeration pond (T3), a biological filter pond (T4), an enhanced purification pond (T5), and a stabilization pond (T6). In the T1 and T2 ponds, emergent plants *Myriophyllum aquaticum* and *Alternanthera philoxeroides* were planted with mature plants, respectively. The submerged plant *Hydrilla verticillata* was used in the T3, T4, and T5 ponds, while *Vallisneria natans* was used in the T6 pond (Figure 1a). During the construction of SFCW, the soaked fruits of *V. natans* and winter buds of *H*. *verticillata* were manually sowed in the corresponding ponds. After a period of growth, these plants fill the pond through asexual reproduction, forming new epiphytic biofilms on the surface. All plants were configured and planted in February 2022, and the SFCW system has been operational since May 2022. The breeding base generates an estimated 10 m^3^/day of aquaculture wastewater. It is discharged into the SFCW through the pipeline from 9 to 12 am every day. Aquaculture wastewater is pumped into the T1 pond, where it undergoes primary sedimentation and absorption before flowing into the T2 pond. Following secondary precipitation and absorption in T2, the water enters the T3 pond, which is aerated for 6 h each day. The treated water then passes through the T4 and T5 ponds before being discharged from the T6 pond. The schematic of the water inlet and outlet is shown in Figure 1b.

Water samples and aquatic plants were collected from the six treatment ponds during July (vigorous growth period of aquatic plant) and November (late growth period of aquatic plant) of 2022. The average precipitation and temperature for both months are provided in Table S1. The biomass data of aquatic plants in different treatment ponds in July and November are provided in Table S2. The water sampling points were located at the inlet and outlet of each treatment pond and were designated as a–f and g (Figure 1b). Three water samples were taken from each of the six stages at both the inlet and outlet, using 500 mL plastic bottles at a depth of 20 cm, in order to assess the role of each stage in nutrient removal. Simultaneously, aquatic plants were collected from the same locations. All plants within a 30 × 30 cm quadrat were harvested, placed in black light-proof plastic bags, and kept on ice packs to prevent biofilm activation.

### 2.2. Water Quality Parameter Determination

Total nitrogen (TN) was determined using alkaline potassium persulfate digestion followed by ultraviolet spectrophotometry (HJ 636-2012 [27]); total phosphorus (TP), total dissolved phosphorus (TDP), and orthophosphate (PO_4_^−^) were measured using ammonium molybdate spectrophotometry (GB 11893-89 [28]) (Ding et al., 2020 [29]; Ross et al., 2022 [30]). Ammonium nitrogen (NH_4_^+^-N), nitrate nitrogen (NO_3_^−^-N), and chemical oxygen demand (COD) were analyzed using a digestion solution with the corresponding parameters and ultraviolet spectrophotometry (DR900, HACH, Loveland, CO, USA) (Cardona et al., 2016 [31]; Xiang et al., 2020 [32]). A YSI portable meter (YSI Incorporated, Yellow Springs, OH, USA) was employed to measure water temperature (T), dissolved oxygen (DO), oxidation-reduction potential (ORP), and pH. The removal efficiency (RE, %) for each treatment pond, as well as the total removal efficiency (TRE, %), were calculated using the following formula:

RE/TRE = (C_in_ × Q_in_ − C_out_ × Q_out_)/(C_in_ × Q_in_) × 100%

where C_in_ and C_out_ represent the wastewater concentrations of the influent and effluent (mg/L), respectively; Q_in_ and Q_out_ represent the daily flow rates of the influent and effluent (m^3^/day), respectively.

### 2.3. Biofilm Extraction Method

After the collected aquatic plants were brought back to the laboratory, surface debris was carefully removed using a fine brush. Stem and leaf tissues with identical shapes, weighing 20 g in total, were placed in a polyethylene bottle and eluted with 100 mL of pre-cooled PBS (50 mM phosphate-buffered saline, pH = 7.4). Following a 5 min ultrasound treatment with the frequency of 40 KHz and subsequent removal of water, the sample was agitated at 225 rpm in a constant-temperature shaker for 10 min before undergoing an additional 3 min ultrasound treatment. The resulting suspensions were collected using a 60-mesh sieve to obtain the plant-attached biofilm containing bacteria. A total of 4 replicate samples were obtained from each plant from each pond for each vegetation period, totaling 48 samples. The biofilm was then harvested and stored at −81 °C in a freezer for subsequent experiments.

### 2.4. Bacterial Sequencing and PCR Amplification

First, the DNeasy PowerBiofilm Pro Kit Rapid DNA extraction kit (item number: 24000, Qiagen, Hilden, Germany) was used to extract genomic DNA from 0.25 g of biofilm, and the extracted DNA was stored at −80 °C in a refrigerator. The extracted genomic DNA was analyzed using 1% agarose gel electrophoresis. Primer pairs 338F (5′-ACTCCTACGGGAGGCAGCAGCAG-3′) and 806R (5′-GGACTACHVGGGTWTCTAAT-3′) were used for the Polymerase Chain Reaction (PCR) tests using AP221-02 TransStart Fastpfu DNA Polymerase (TransGen Biotech, Beijing, China) in a reaction volume of 20 μL. The reaction conditions were as follows: 95 °C for 3 min; 24 cycles of 95 °C for 30 s, 50 °C for 30 s, 72 °C for 45 s; and a final step at 72 °C for 10 min. The PCR products were mixed and detected using 2% agarose gel electrophoresis, followed by recovery using the AxyPrep DNA Gel Recovery Kit (Axygen Biosciences, Union City, CA, USA). The PCR products were quantified using the QuantiFluor™-ST blue fluorescence quantification system (Promega, Madison, WI, USA) with reference to the initial electrophoresis quantification results and then mixed according to the sequencing volume requirements of each sample. All samples were sequenced by MajorBio (Shanghai, China) using the Illumina MiSeq platform. Finally, the 16S rDNA sequences were compared with reference sequences in the SILVA database (http://www.arb-silva.de, accessed on 20 July 2023) to identify the different species in different groups on the biofilm samples based on clustering of operational taxonomic units (OTUs) at a 97% similarity level.

### 2.5. Statistical Analyses

The Wilcoxon test was used to determine differences in water parameters between the two sampling periods. Differences in 16S rRNA gene abundance and bacterial alpha diversity among different groups and periods were analyzed using one-way/two-way analysis of variance (ANOVA). The ‘vegan’ package in R was used for nonmetric multidimensional scaling analysis (NMDS) and analysis of permutational multivariate ANOVA (PERMANOVA), and the Bray–Curtis distance was used to calculate the beta diversity matrix to detect the changes in attached bacteria at the OTU level. Redundancy discriminant analysis (RDA) and Monte Carlo permutation tests (permutations = 999) were used to analyze the effects of environmental factors on bacterial community structure using the ‘vegan’ package (Oksanen et al., 2016 [33]). All the above analyses were performed in R (version 4.2.2; http://www.r-project.org/, accessed on 25 August 2024). To analyze the energy metabolism functions of bacteria, PICRUSt2 was used to predict bacterial function (Douglas et al., 2020 [34]). In addition, bacterial function was analyzed using the STAMP software (version 2.1.3) (Parks et al., 2014 [35]).

## 3. Results

### 3.1. Performance of the Multi-Stage SFCW

Our study reveals changes in pollutant concentrations in wastewater entering the multi-stage SFCW system between July and November (Table S3). Specifically, the concentrations of TN and TP in the wastewater entering the SFCW were 2.4 and 4.2 times higher, respectively, in July compared to November. Consequently, concentrations of TDP, PO_4_^−^, NH_4_^+^-N, and NO_3_^−^N were generally higher in July than in November. However, the concentration of COD was notably higher in November than in July. Notably, we found that the concentration of NH_3_^+^-N in the treatment ponds exceeded that of NO_3_^−^N in July, while the reverse was true in November (Table S3).

**Figure 2:**
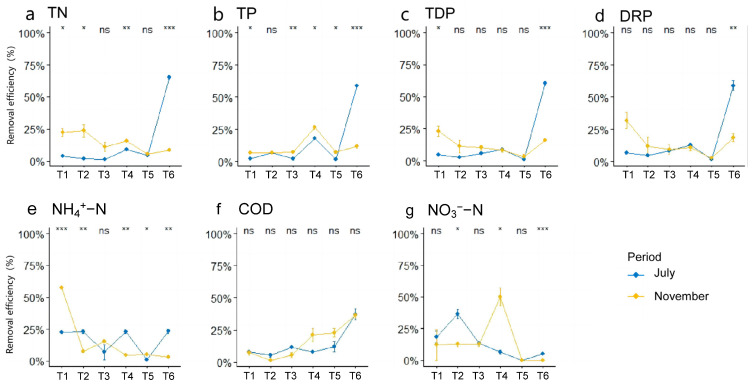
The contribution of each treatment pond to pollutant removal. TN, total nitrogen; TDP, total dissolved phosphorus; COD, chemical oxygen demand; TP, total phosphorus; PO_4_^−^, orthophosphate; NO_3_^−^-N, nitrate nitrogen; NH_4_^+^-N, ammonia nitrogen. The asterisk indicates a significant difference between the removal efficiency of the same treatment pond in July and November (Wilcoxon test); * *p* < 0.05; ** *p* < 0.01; *** *p* < 0.001, ns: not significant.

The contribution of each treatment pond to pollutant removal efficiency (RE) is presented in Figure 2. In July, the RE of TN was lower in T1 to T5, with the highest RE observed in T6, reaching 65.49% (Figure 2a). In comparison to T1, T2, and T3, the RE of TP increased in T4 but decreased in T5, reaching its peak value of 58.54% in T6 (Figure 2b). The variation trends for TDP and PO_4_^3−^ followed similar patterns to TN, with the highest RE observed in T6 (Figure 2c,d). The RE of NH_4_^+^-N fluctuated significantly in July, with the lowest RE in T5 and the highest in T6 (Figure 2e). The RE of COD exhibited an increasing trend from T1 to T6, peaking at 37.21% in T6 (Figure 2f). In contrast, the RE of NO_3_^−^-N showed a decreasing trend, reaching its highest RE in T2 (Figure 2g). In November, the RE of TN in all treatment ponds (T1 to T6) remained below 25%, showing a decreasing trend across the stages (Figure 2a). The RE of TP from T1 to T5 was similar to July, but the peak occurred in T4 (rather than T6), reaching 26.50% (Figure 2b). The variation trends for TDP and PO_4_^−^ followed similar patterns, with the highest RE observed in T1 and the lowest in T6 (Figure 2c,d). The RE of NH_4_^+^-N showed a decreasing trend from T1 to T6, with the highest RE in T1, reaching 57.35% (Figure 2e). In contrast, the RE of COD increased across the treatment ponds, with the highest RE in T6 (Figure 2f). The RE of NO_3_^−^-N was highest in T4, while the RE in the other treatment ponds was relatively low (Figure 2g). Among the six treatment ponds, pollutant removal efficiency in T6 was most significantly affected between the two sampling periods, July and November (*p* < 0.05).

**Figure 3:**
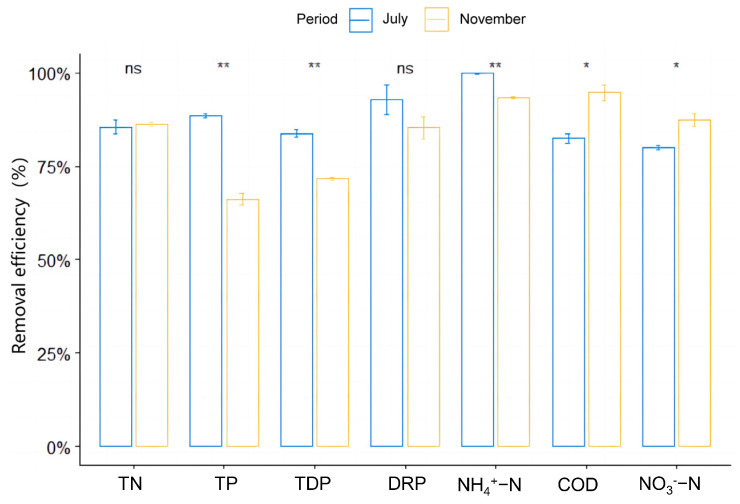
Total removal efficiency of pollutants in the SFCW system in July and November. Asterisks indicate a significant difference between July and November; (Wilcoxon test); * *p* < 0.05; ** *p* < 0.01; ns: not significant.

The total removal efficiency (TRE) of the SFCW system is presented in Figure 3. The TRE exceeded 60% in both July and November. The TRE of TP, TDP, and NH_4_^+^-N was significantly higher in July compared to November (*p* < 0.05), whereas the TRE of COD and NO_3_^−^-N was significantly higher in November than in July (*p* < 0.05). However, no significant differences were observed between the two vegetation periods in the TRE of TN and PO_4_^−^ (*p* > 0.05).

### 3.2. Abundance and Alpha Diversity of Epiphytic Biofilm

A total of 3,091,704 valid, readable sequences were obtained from 48 biofilm samples. After quality control and purification, 2,384,855 unique representative sequences were generated, resulting in a total of 7908 OTUs. The values for observed species, Chao1, and the Shannon index of epiphytic biofilm samples from the T2–T6 ponds were all significantly lower in July compared to November (*p* < 0.05) (Figure S1). In contrast, the observed species and Chao1 values for the epiphytic biofilm in the T1 pond were higher in July than in November. Additionally, both the observed species, Chao1, and Shannon index of the epiphytic biofilm were highest in the T3 pond and lowest in the T2 pond during both periods (Figure S1).

### 3.3. Bacterial Community Composition and Structure Variation in Epiphytic Biofilm

**Figure 4:**
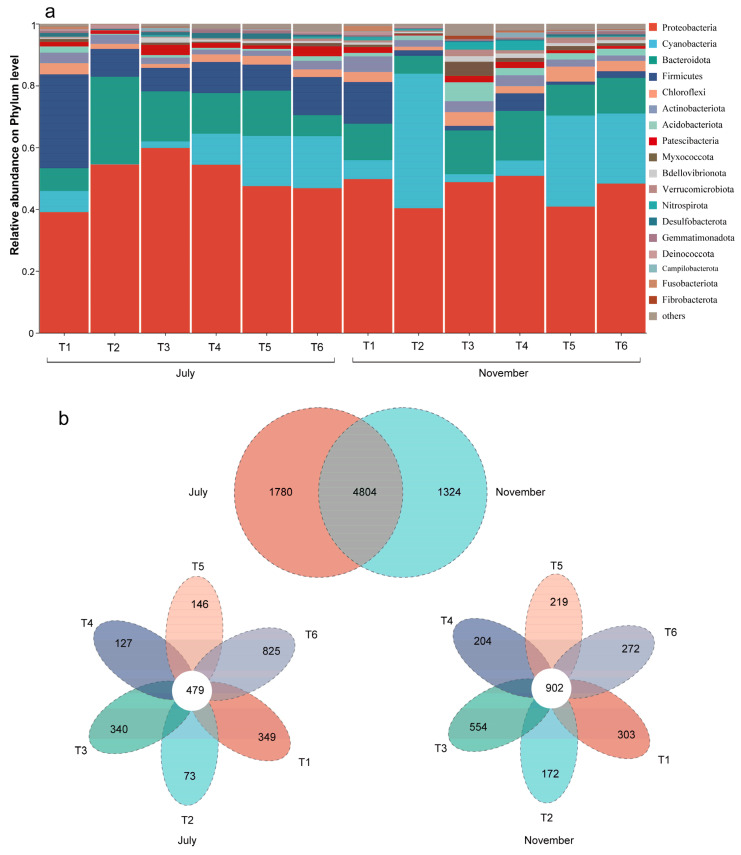
Community compositions of bacteria (relative abundance > 1%) at the phylum level across different treatment ponds in July and November (**a**). Venn diagram of epiphytic bacterial OTUs based on different treatment ponds in July and November (**b**).

A total of 56 phyla, 165 classes, 376 orders, 645 families, and 1267 genera were detected in the 48 biofilm samples. Proteobacteria (39.1–59.8%) was the dominant phylum in the epiphytic biofilm samples. Other dominant phyla included Cyanobacteria (2.1–43.6%), Bacteroidota (5.9–28.2%), and Firmicutes (1.0–30.4%), although their relative abundances varied significantly between two periods and treatment ponds (Figure 4a). Among the epiphytic bacteria, Cyanobacteria, Acidobacteriota, and Myxococcota were significantly more abundant in November than in July, whereas Firmicutes exhibited the opposite pattern (Figure S2). In both July and November, the relative abundance of Firmicutes in the T1 pond was significantly higher than in the other ponds. Additionally, the highest abundance of Cyanobacteria was observed in the T2 pond in November, while the highest abundance of Bacteroidota was found in the T2 pond in July (Figure 4a). A Venn diagram based on OTUs was used to estimate the common and unique species across different periods and treatment ponds (Figure 4b). In terms of vegetation period differences, 1780 unique OTUs were recorded in July, while 1324 unique OTUs were recorded in November. Moreover, the number of unique OTUs in the epiphytic biofilms of T1 and T6 ponds decreased in November compared to July, while the other treatment ponds showed varying degrees of increase (Figure 4b).

**Figure 5:**
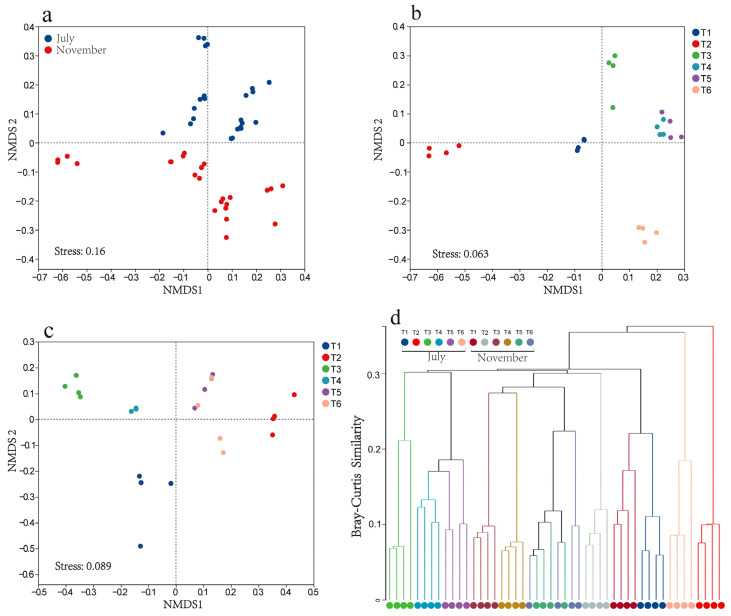
Nonmetric multidimensional scaling diagram showed the differences in epiphytic bacterial community structure at the OTUs level (calculated using Bray–Curtis) between two periods (**a**), six treatment ponds in July (**b**) and six treatment ponds in November (**c**). Hierarchical clustering of the samples based on Bray–Curtis similarity and the average sample on OTU level (**d**).

Non-metric multidimensional scaling (NMDS) based on Bray–Curtis similarity was employed to further examine the differences in the community structure of epiphytic bacteria across various treatment ponds during two periods. The results showed that the composition of the bacterial community varied significantly during different vegetation periods (Figure 5a). Additionally, notable differences in the epiphytic biofilm community structure were observed between the different treatment ponds (Figure 5b,c), a finding further corroborated by hierarchical clustering analysis based on Bray–Curtis similarity (Figure 5d). Furthermore, the PERMANOVA analysis indicated that the community structure of epiphytic bacteria was significantly influenced by both the period (R^2^ = 0.1589) and treatment pond (R^2^ = 0.3897), with the effect of the treatment pond being more pronounced (Table 1).

### 3.4. Relationship Between Bacterial Community and Environment

**Figure 6:**
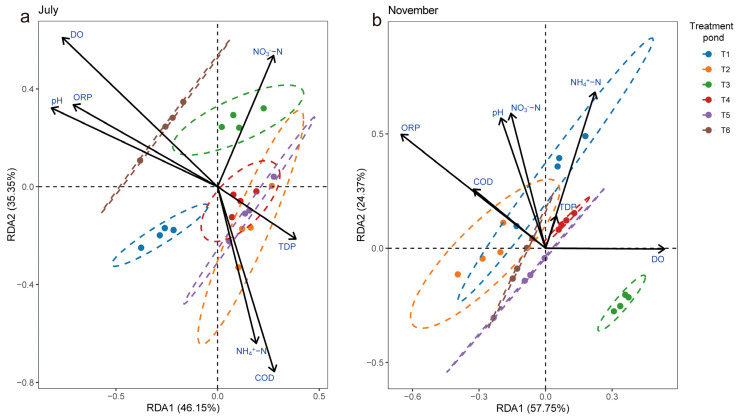
Redundancy analysis of the bacterial community structure and environmental variables in the epiphytic bacteria in July (**a**) and November (**b**). TN, total nitrogen; TDP, total dissolved phosphorus; COD, chemical oxygen demand; TP, total phosphorus; PO_4_^−^, orthophosphate; NO_3_^−^-N, nitrate nitrogen; NH_4_^+^-N, ammonia nitrogen; pH, water pH; DO, dissolved oxygen; ORP, oxidation reduction potential.

Redundancy analysis revealed that 87.28%, 81.5%, and 82.12% of the overall variability in both periods and of the bacterial community composition in July and November, respectively, were explained by the first two principal components, RDA1 and RDA2 (Figure S4 and Figure 6). When assessing the physical and chemical factors of the water in different periods, the dynamics of the biofilm bacterial community were found to be mainly driven by T, TN, PO_4_^−^, NO_3_^−^-N, COD, and DO (*p* < 0.05). Water temperature (R^2^ = 0.117, *p* = 0.001) was the most significant variable affecting the bacterial community in the different periods, whereas TP, TDP, NH_4_^+^-N, pH, and ORP showed relatively little correlation with the bacterial community (*p* > 0.05) (Figure S4). Furthermore, based on redundancy analysis, we found that DO (R^2^ = 0.113, *p* = 0.001) was the most significant variable affecting bacterial communities in July, followed by pH, TDP, NO_3_^−^-N, and NH_4_^+^-N (Figure 6a). We found that the variance in November bacterial community dynamics was mainly driven by factors such as NO_3_^−^-N, NH_4_^+^-N, pH, COD, and ORP (Figure 6b).

### 3.5. Prediction of Bacterial Function

**Figure 7:**
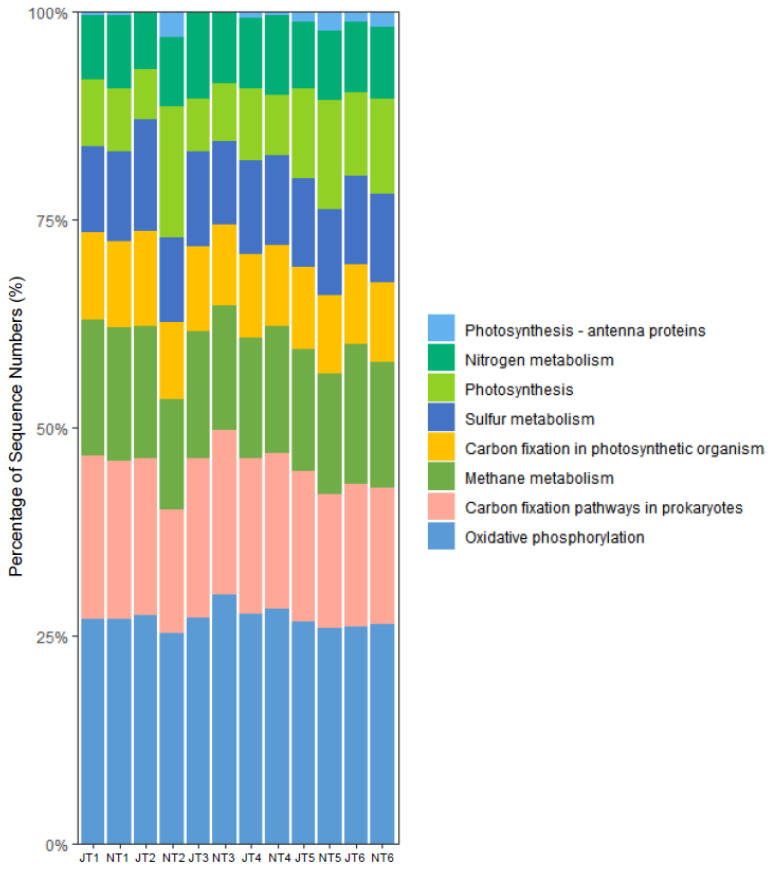
The bar chart of bacterial energy metabolism function predicted by different ponds in two vegetation periods at the level of the second KEGG pathway. T1 to T6 represent the six treatment ponds, J for July and N for November.

To explore the role of energy metabolism in bacterial communities, we used PICRUSt2 to predict the function of 16S rRNA sequences and compared the predicted functions at the energy metabolism level (Figure 7). The overall comparison between two vegetation periods showed that the abundance of photosynthesis and sulfur metabolic pathway functional genes in epiphytic bacteria were significantly higher in July than in November (*p* < 0.05) (Figure S5a). A comparison of individual treatment ponds in different periods showed that there was no significant difference in the abundance of functional genes for energy metabolic pathways of attached bacteria in T1, T4, T5, and T6 in July or November (*p* > 0.05). However, the abundance of functional genes for the carbon fixation, sulfur metabolism, methane metabolism, and oxidative phosphorylation pathways of attached bacteria in T2 was significantly higher in July than in November (*p* < 0.05), whereas the abundance of photosynthesis and nitrogen pathway functional genes was significantly higher in November than in July (*p* < 0.05) (Figure S5b). In T3, the abundance of oxidative phosphorylation and prokaryotic carbon metabolic pathway functional genes was significantly higher in November than in July (*p* < 0.05), and the abundance of sulfur metabolism and photosynthetic carbon fixation pathway functional genes was significantly higher in July than in November (*p* < 0.05) (Figure S5c).

## 4. Discussion

Wastewater from aquaculture is typically characterized by elevated nutrient levels, with nitrogen and phosphorus being key contributors to environmental degradation (Kurniawan et al., 2021 [1]). In our study, the concentration of influent pollutants was significantly higher in July compared to November. This discrepancy can be attributed to the breeding cycle of the bullfrog, where July represents the peak feeding period, leading to an increased concentration of wastewater, while November marks the conclusion of the breeding season. Despite the concentration of pollutants entering the SFCW varying over time, the TRE for TN remained relatively stable, whereas the TRE for TP was notably higher in July. These findings suggest that the treatment performance of the SFCW is more consistent in removing TN. Previous studies have indicated that nitrogen removal in constructed wetlands occurs primarily through processes such as ammonification, nitrification, and denitrification by microorganisms. Among these, denitrification plays a crucial role by converting nitrates into nitrogen gas, which is then released into the atmosphere (Li et al., 2019 [36]; Tang et al., 2020 [37]). The multi-stage environment in SFCWs provides diverse redox conditions, enhancing the stability of total nitrogen removal (Wu et al., 2015 [38]). In contrast, phosphorus removal is primarily driven by substrate adsorption, precipitation, nutrient uptake by aquatic plants, and microbial activity within the wetland (Abdoli et al., 2024 [39]; Li et al., 2020 [40]). In July, when aquatic plants are actively growing and microbial activity is at its peak (Li et al., 2019 [41]), the system demonstrates higher removal efficiency for TP compared to November. Moreover, our results demonstrate that the SFCW system can achieve over 60% removal of various pollutants in both periods. These favorable TRE values highlight the system’s effectiveness in intercepting nutrients, contributing significantly to the reduction in the pollution load in Lake Datong.

Additionally, we observed variations in the contributions of different treatment ponds to pollutant removal. Previous studies have demonstrated that water treatment processes can significantly enhance pollutant removal efficiency (Nidheesh et al., 2021 [42]). In our study, we found that when wastewater passed through the aeration pond (T3), the RE of TDP, PO_4_^−^, and COD increased slightly in July, while the RE of NH_4_^+^-N and COD also improved in November, with NH_4_^+^-N showing the most pronounced change. These variations in pollutant removal efficiency are likely associated with increased levels of DO. Our results indicated that T3 had the highest DO content among all treatment ponds during both periods. DO is a critical limiting factor in nitrogen removal, as it not only directly influences the growth of nitrifying and denitrifying bacteria but also affects the concentration of organic carbon in influent water, thereby indirectly impacting denitrification processes (Lu et al., 2020 [43]; Vymazal, 2007 [44]). Furthermore, elevated DO levels can enhance the abundance of phosphorus-accumulating bacteria, thereby improving the conversion and absorption of dissolved phosphate from the water (Zhao et al., 2017 [45]). Artificial aeration is widely regarded as the most direct and effective method for supplementing DO in constructed wetlands, as it can improve the oxygen transfer rate and significantly enhance the pollutant removal performance in aerated constructed wetlands (Wu et al., 2014 [46]).

The results indicated that the RE of TP and TN increased in both July and November during the passage of wastewater through the biological filter (T4). Previous studies have shown that biological filters primarily rely on the physiological and metabolic functions of microorganisms to transform pollutants, such as nitrogen and phosphorus, into forms that are assimilated by microorganisms or plants, thereby achieving purification effects (Schreier et al., 2010 [47]; Wu et al., 2018 [48]). Additionally, the filter material layer in biological filters provides a substantial surface area for microorganisms to attach and proliferate. The synergistic interactions among various microorganisms within the biofilm can lead to the extensive degradation of pollutants (Ding et al., 2023 [49]). From the perspective of the aquatic plant epiphytic biofilm diversity in T4, which ranks second only to T3, we hypothesize that the diversity of the biofilm in the filter material layer is likely high. The transformation of the biofilm within the filter layer, combined with the nutrient absorption by aquatic plants, may enhance the overall performance of the biological filter (Irhayyim et al., 2021 [50]), thereby improving the RE of nitrogen and phosphorus in wastewater. Furthermore, our results showed that the RE of NH_4_^+^-N increased significantly in July, while the removal efficiency of NO_3_^−^-N was notably higher in November. Temperature is a critical factor affecting the performance of constructed wetlands, with both aquatic plants and nitrogen-transforming bacteria exhibiting temperature-dependent patterns (Peng et al., 2014 [51]). In July, favorable temperatures promote the active growth of aquatic plants, leading to high DO concentrations, which, in turn, enhance biofilm activity and facilitate nitrification, thereby promoting the removal of NH_4_^+^-N. However, the relatively lower temperatures and DO levels in November are more conducive to the denitrification process, where NO_3_^−^-N is reduced to nitrogen gas. Moreover, our results showed that the abundance of Nitrospirota was significantly higher in November compared to July. Previous studies have demonstrated that Nitrospirota can efficiently enhance NO_3_^−^-N removal by utilizing nitrate as a nitrogen source through the assimilative nitrate reduction pathway (Zhao et al., 2023 [52]). Therefore, we speculate that the increased removal of NO_3_^−^-N in November may be attributed to the higher abundance of Nitrospirota.

The RE of pollutants in the same treatment pond varied significantly between two vegetation periods, particularly in the stabilization pond (T6). These variations are likely related to differences in plant species and seasonal changes (Vymazal, 2011 [53]). Previous studies have indicated that plant species are the primary factors influencing the effectiveness of water restoration, as the growth rates and nutrient absorption capacities of different plant species vary considerably (Zhang et al., 2022 [54]). In the present study, the RE of nitrogen and phosphorus in T6, which was planted with the submerged plant *V. natans*, was highest in July. This can be attributed to the vigorous absorption and utilization of nitrogen and phosphorus by the leaves and roots of *V. natans* during its active growth period (Shen et al., 2023 [55]). However, in November, the RE of nitrogen and phosphorus in T6 decreased significantly, likely due to seasonal changes that indirectly affected the absorption capacity of submerged plants by influencing temperature and growth conditions (Li et al., 2020a [56]). Additionally, we observed that the pollutant removal rate in treatment ponds planted with emergent plants (e.g., T1, T2) did not show significant seasonal variation, although an increase was noted in November. We speculate that while emergent plants may not absorb pollutants as effectively as submerged plants, they may exhibit greater stability in response to seasonal changes.

Aquatic plants play a crucial role in shaping the composition of bacterial communities in aquatic ecosystems (Dai et al., 2019 [57]). Distinct chemical and physiological processes are associated with different life forms of aquatic plants, and host-specific epiphytic bacterial communities are influenced by the activities of these plants, leading to variations in the bacterial community composition across different plant species (He et al., 2012 [58]; Hempel et al., 2008 [59]; Rich et al., 2012 [60]). In our study, the bacterial diversity of submerged plants (T3, T4, T5, and T6) was higher than that of emergent plants (T1 and T2) (Figure 4), likely due to the greater surface area and higher DO levels provided by submerged plants, which facilitate enhanced bacterial colonization (Pettit et al., 2016 [61]). Furthermore, we observed a gradual decline in bacterial diversity from T3 to T6, which may be attributed to the decreasing concentrations of pollutants. Previous studies have indicated that phosphorus and nitrogen are key limiting factors for bacterial growth, and a reduction in nutrient levels can influence the selection of host plants by bacteria (Liu et al., 2015 [62]). The results of the RDA also highlighted that nutrients, such as TN and TP, were the primary factors driving changes in microbial communities within the epiphytic biofilm. As the treatment process progresses, the pollutant content in T3 to T6 gradually decreases, which may reduce the bacterial selection pressure exerted by host plants, resulting in a gradual decline in bacterial diversity. Additionally, the observed species, Chao1, and Shannon diversity indices of epiphytic biofilm samples in the T2-T6 ponds were all significantly lower in July compared to November. Similar patterns have been reported by Yu et al. (2022) [63]. The variation in the epiphytic bacterial community composition may be linked to the life cycle of host plants, leaf morphology, and the characteristics of surrounding water (Korlevic et al., 2021 [64]). Moreover, as planktonic bacteria continue to adsorb and adhere to the surfaces, more mature biofilms form in November, which likely contributes to the higher diversity of the apparent biofilm in November compared to July (Kimkes and Heinemann, 2020 [65]).

The composition and structure of bacterial communities in aquatic environments are often influenced by environmental conditions, and seasonal variations can significantly affect the composition of epiphytic bacterial communities on aquatic plants (Cole et al., 2013 [66]). For instance, temperature can directly impact the structure of bacterial communities in epiphytic biofilms, as well as indirectly affect them through its influence on the physiological activities of aquatic plants (Korlevic et al., 2021 [64]). Our RDA results further confirm that temperature is the most significant variable influencing both July and November epiphytic bacterial communities. However, our findings also indicated that the vegetation period and treatment pond significantly affected the community structure of epiphytic bacteria, with the treatment pond having a more pronounced effect. These results suggest that the environmental heterogeneity induced by different treatment processes in our study has a more substantial impact on the structure of biofilm communities. Previous studies have shown that treatment processes such as aeration can increase the concentration of dissolved oxygen (DO), alter the composition of bacterial communities in attached biofilms, and enhance biofilm activity and diversity (Lai et al., 2020 [67]; Xu et al., 2023 [68]). Additionally, the filter material and baffle configuration in biological filter ponds can increase the surface area available for bacterial attachment, thereby facilitating the colonization of more bacteria and altering the bacterial community composition (Ren et al., 2023 [69]). Moreover, differences in the species of aquatic plants planted in different treatment ponds, as well as variations in influent concentrations, can also contribute to differences in the structure of epiphytic bacterial communities (Saeed et al., 2023 [70]).

Functional genes are key bacterial indicators that drive the metabolic cycling of environmental factors (Zhou et al., 2022 [71]). In our study, the overall functional characteristics of the bacterial communities were similar across different samples. Both oxidative phosphorylation and carbon fixation pathways were enriched in all samples, accounting for more than 15% of the energy metabolic pathways. As core energy metabolism pathways, these two processes were the primary drivers of microbial community metabolism and activities within the constructed wetland (Ren et al., 2017 [72]). Additionally, bacterial communities were enriched in various metabolic pathways related to exogenous substances, such as nitrogen and sulfur metabolism-, highlighting the pivotal role of bacterial communities in the self-purification and remediation of exogenous pollutants in aquatic ecosystems (Zhang et al., 2021 [73]). The metabolic performance of bacteria in epiphytic biofilms is closely linked to the composition and structure of the bacterial community (Besemer, 2015 [74]; Goodchild-Michelman et al., 2023 [75]). In this study, the abundance of functional genes related to carbon fixation and sulfur metabolism was significantly lower in November compared to July. Previous studies have indicated that the abundance of functional genes typically correlates with bacterial abundance (Li et al., 2023 [76]). Among the bacterial taxa that exhibited decreased abundance in November, Firmicutes are involved in sulfur metabolism, Proteobacteria are linked to sulfate reduction, and Bacteroidota play a crucial role in the carbon cycle (Gupta et al., 2018 [77]; Ma et al., 2023 [78]; Zhou et al., 2020 [79]). Therefore, as the abundances of Firmicutes, Proteobacteria, and Bacteroidota decreased, there was a corresponding reduction in the abundance of functional genes involved in carbon fixation and sulfur metabolism. Furthermore, the functional genes of the secondary sedimentation pond (T2) and aeration pond (T3) were also different in different vegetation periods. Bacteria adapt to various environmental stressors by altering gene expression (Zhang et al., 2020 [80]). We speculated that, in November, under nutrient-limited conditions in T2, bacteria may downregulate the expression of non-essential functional genes, such as those involved in carbon fixation and sulfur metabolism, to conserve energy and adapt to the environment. Concurrently, the expression of photosynthesis-related genes may be upregulated to compensate for the energy gap (Xu et al., 2022 [81]). Moreover, we observed that functional genes associated with nitrogen metabolism in T2 were significantly more abundant in November than in July, and the RE of TN in T2 was also significantly higher in November. These findings suggest that the changes in the abundance of functional genes can be directly reflected in the water treatment efficiency.

## 5. Conclusions

The multi-stage SFCW system effectively intercepts various pollutants in aquaculture wastewater. With the change of time, the performance of the multi-stage constructed wetland in treating total nitrogen becomes more stable. The integration of treatment processes, such as aeration and biological filters, significantly enhances the overall performance of constructed wetlands. However, treatment ponds with submerged plants experience a notable decrease in performance during November, which can be attributed to the life cycle of submerged plants. The alpha diversity of epiphytic bacteria was significantly higher in November than in July. Additionally, the abundance of Firmicutes was notably greater in November compared to July, while Nitrospirota and Acidobacteriota showed significantly higher abundances in November than in July. Among the different treatment ponds, the alpha diversity of epiphytic bacteria in the aeration pond (T3) and biological filter pond (T4) was significantly higher than in the other treatment ponds, indicating that these treatment processes promote the growth of epiphytic bacteria. The results of the PICRUSt2 analysis indicated that both growth period variations and treatment processes can influence the predicted functional potential of epiphytic bacterial communities based on inferred gene abundances. In summary, when designing constructed wetlands for aquaculture wastewater treatment, increasing the number of stabilization ponds and incorporating appropriate aeration devices and biofilters can significantly enhance overall treatment performance. This study also provides valuable insights into the structural and functional changes of epiphytic biofilms in constructed wetlands.

## Figures and Tables

**Table 1:** The result of permutation-based multivariate analysis of variance of epiphytic bacterial community compositions. The factors of vegetation period and treatment pond were put together to check their significance in causing bacterial community composition changes (reflected in Bray–Curtis similarity among different samples). The significant effects are shown in bold (*p* < 0.05).

	Df	Sums of Squares	Mean Squares	Pseudo-*F*	*R* ^2^	***p***
Vegetation periods	1	2.02397	2.02397	14.42954	0.1589	**0.001**
Treatment ponds	5	4.96508	0.99302	7.07953	0.3897	**0.001**
Residuals	41	5.7509	0.14027		0.4514	
Total	47	12.73995			1	

## Data Availability

The original contributions presented in this study are included in the article/Supplementary Material. Further inquiries can be directed to the corresponding author.

## Supplementary Materials

The following supporting information can be downloaded at: https://www.mdpi.com/article/10.3390/microorganisms13030494/s1.
