# Supplementary material for: The Performance of a Multi-Stage Surface Flow Constructed Wetland for the Treatment of Aquaculture Wastewater and Changes in Epiphytic Biofilm Formation
Source: Microorganisms. 2025 Feb 22;13(3):494. doi: 10.3390/microorganisms13030494 (PMC11944938; doi:10.3390/microorganisms13030494)
Supplement: Supplementary file 1 [file microorganisms-13-00494-s001.zip › microorganisms-3427327-supplementary.pdf]

Supplementary Figures

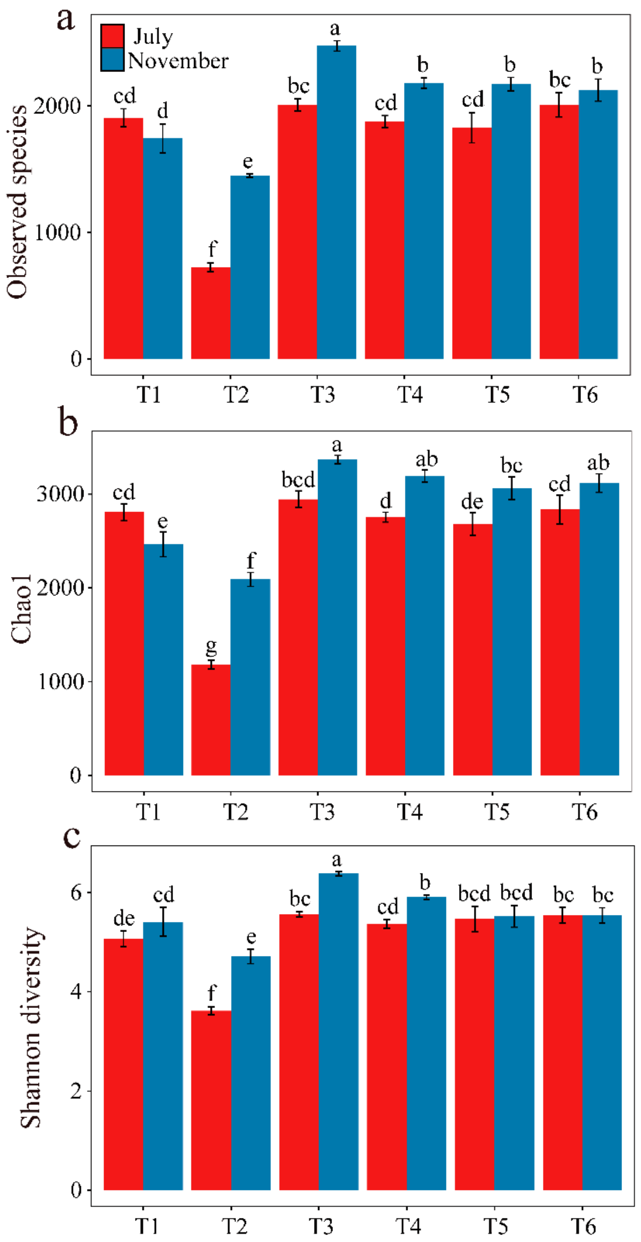

Figure S1. Alpha diversity estimates of epiphytic biofilm in six treatment ponds in July and November. The observed species (a), Chao1 (b) and Shannon index(c). Different letters (a, b, c, d, e, f) represent significant differences ($P < 0.05$) in mean value by means of two-way ANOVAs and LSD post-hoc comparisons.

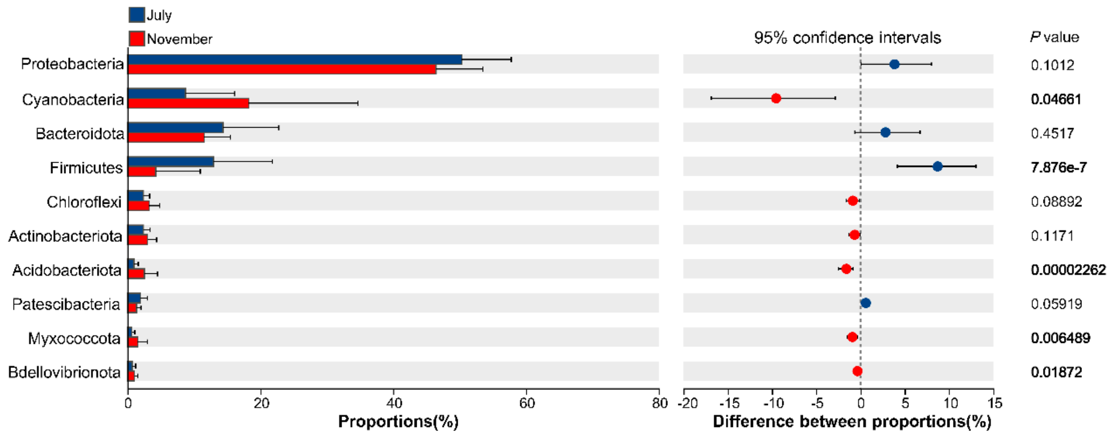

Figure S2. Abundance difference of dominant phylum (top 10) in July and November.

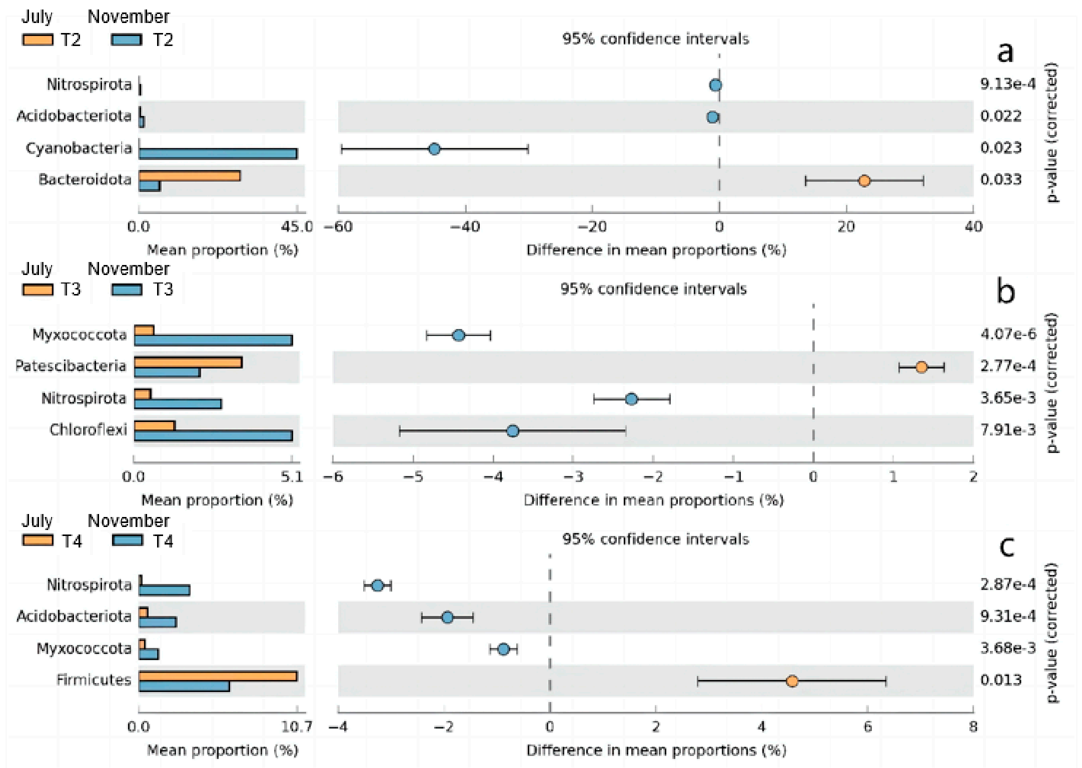

Figure S3. Abundance difference of dominant phylum in secondary sedimentation pond (T2) (a), aeration pond (T3) (b) and biological filter pond (T4) (c) between July and November.

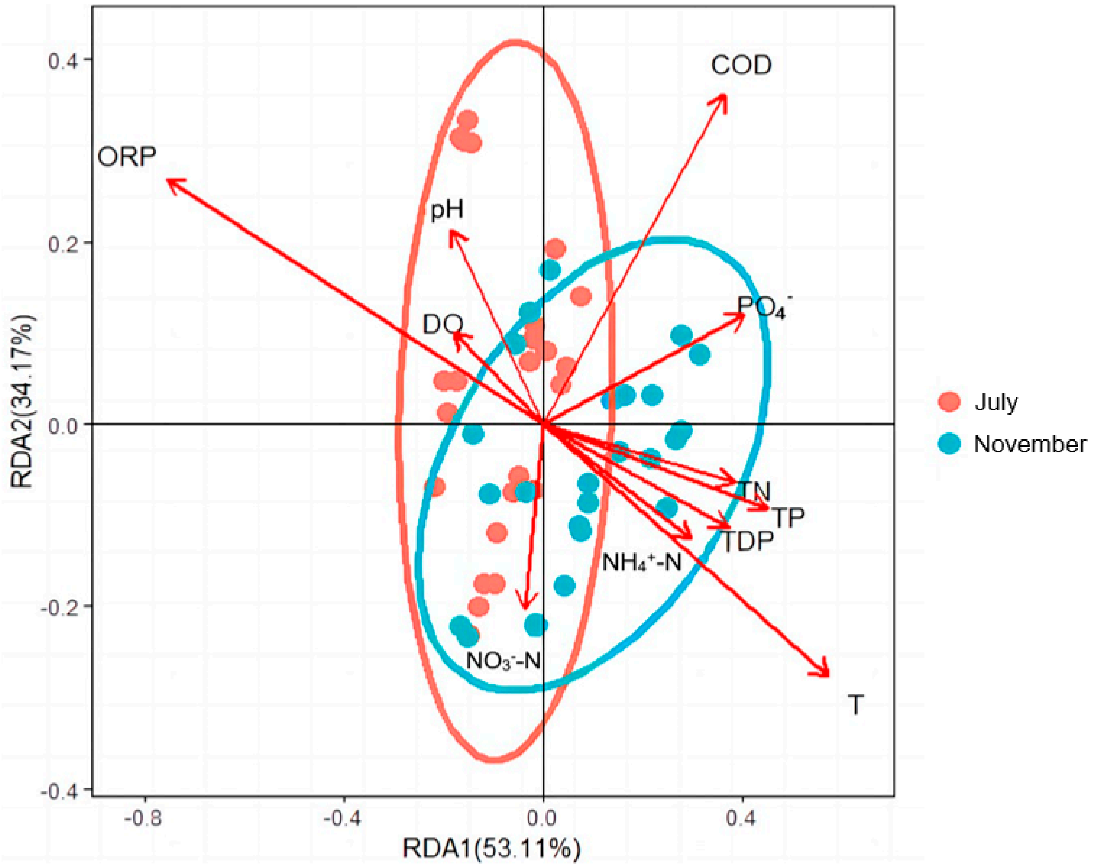

Figure S4. Redundancy analysis of the bacterial community structure and environmental variables in the July (a) and November (b). TN, total nitrogen; TDP, total dissolved phosphorus; COD, chemical oxygen demand; TP, total phosphorus; PO_4^-, orthophosphate; NO_3^-, nitrate nitrogen; NH_3^+-N, ammonia nitrogen; pH, water pH; DO, dissolved oxygen; ORP, oxidation reduction potential.

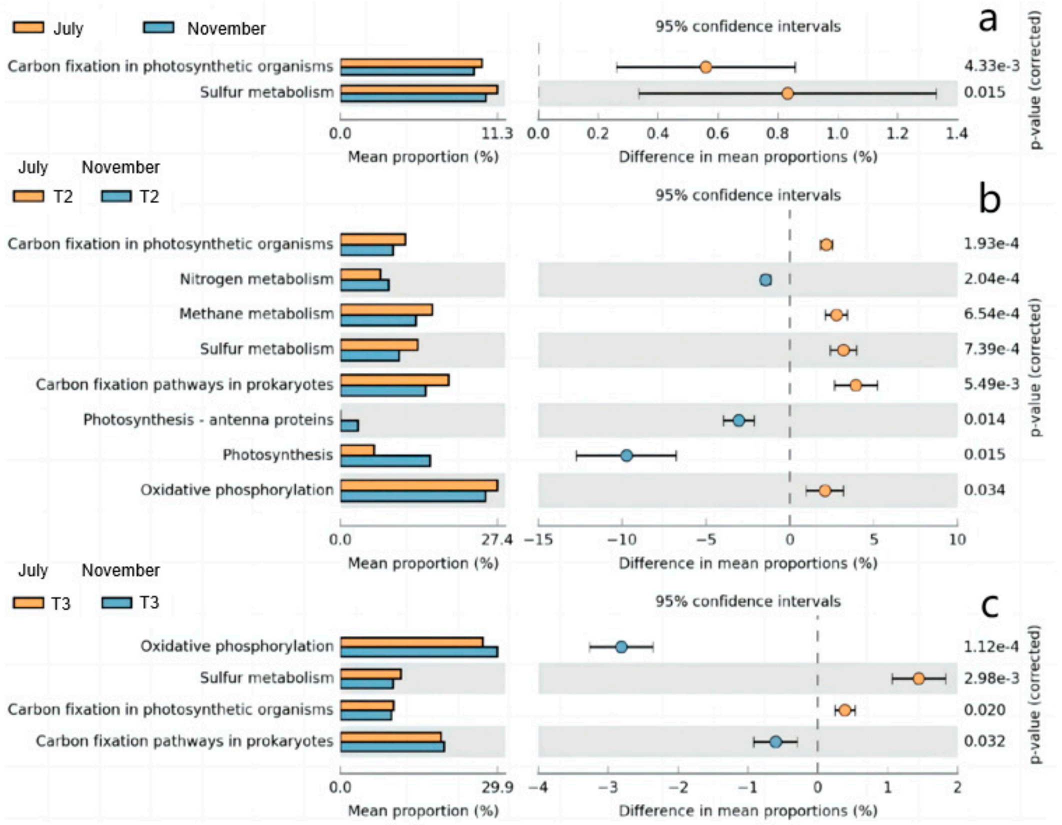

Figure S5. Differences of energy metabolism functions of epiphytic bacteria in July and November (a), secondary sedimentation pond (T2) (b) and aeration tank (T3) (c) between July and November.

Supplementary Table

Table S1 Average temperature and precipitation for July and November 2022

	Temperature (°C)	Precipitation (mm)
July	31.3±1.6	116±12.3
November	8.5±1.2	18±3.2

Table S2 Biomass (g/m^2) of aquatic plants in different treatment ponds during two growth periods in July and November.

Ponds	July	November
T1	1398.4±22.6	1392.4±25.6
T2	3067.4±17.9	2885.2±21.6
T3	1023.7±30.6	1436.1±17.4
T4	1638.6±28.5	1635.1±23.5
T5	260.1±18.4	2361.7±29.4
T6	550.4±17.8	528.9±20.7

Table S3 Physical and chemical parameters of water among six treatment tanks in July and November. TN, total nitrogen; TP, total phosphorus; TDP, total dissolved phosphorus; PO_4^-, orthophosphate; NH_3^+-N, ammonia nitrogen; NO_3^-, nitrate nitrogen; COD, chemical oxygen demand; DO, dissolved oxygen; pH, water pH; T, water temperature; ORP, oxidation reduction potential. Lowercase letters next to numbers represent significant differences (*P* < 0.05) between different ponds.

Parameters	July						
	a	b	c	d	e	f	g
TN (mgL^{-1})	5.73±0.08^{a}	5.51±0.07^{a}	5.41±0.02^{ab}	5.34±0.05^{ac}	4.83±0.08^{de}	4.58±0.09^{e}	0.83±0.19^{f}
TP (mgL^{-1})	0.72±0.01^{a}	0.71±0.01^{b}	0.66±0.01^{c}	0.65±0.01^{d}	0.52±0.01^{e}	0.51±0.01^{e}	0.08±0.01^{f}
TDP (mgL^{-1})	0.62±0.01^{a}	0.59±0.01^{b}	0.57±0.01^{c}	0.54±0.01^{d}	0.48±0.01^{e}	0.48±0.01^{e}	0.1±0.01^{f}
DRP(mgL^{-1})	0.3±0.01^{a}	0.28±0.01^{b}	0.27±0.01^{b}	0.24±0.01^{c}	0.2±0.01^{d}	0.2±0.01^{d}	0.02±0.02^{e}
NH_3^+-N(mgL^{-1})	4.37±0.59^{a}	3.39±0.54^{b}	2.39±0.53^{c}	2.05±0.02^{c}	1.05±0.01^{d}	1.02±0.01^{d}	0.07±0.03^{e}
NO_3^-(mgL^{-1})	2±0.1^{a}	1.63±0.23^{b}	0.9±0.1^{c}	0.63±0.06^{d}	0.5±0.01^{de}	0.5±0.01^{de}	0.4±0.01^{e}
COD (mgL^{-1})	36.67±1.53^{a}	33.67±1.53^{ab}	31.67±0.58^{b}	27.33±0.58^{c}	24.33±0.58^{c}	20±2.65^{d}	6.4±4.13^{e}
DO (mgL^{-1})	3.67±0.02^{c}	3.3±0.17^{c}	0.13±0^{f}	5.5±0.01^{b}	5.29±0.02^{bc}	0.52±0.04^{d}	6.27±0.08^{a}
pH	8.43±0.12^{a}	8.37±0.19^{a}	8.28±0.22^{a}	8.43±0.41^{a}	8.2±0.09^{a}	8.3±0.14^{a}	8.44±0.21^{a}
T (°C)	30.53±0.15^{b}	31±1^{b}	30.7±0.2^{b}	30.47±0.25^{b}	30.47±0.15^{b}	30.27±1.04^{b}	33.7±0.01^{a}
ORP (mV)	199.07±2.03^{a}	193.23±7.16^{a}	-16.2±1.49^{d}	141.63±3.61^{b}	58.97±3.2^{c}	-87.23±1.27^{e}	138.43±1.53^{b}

Parameters	November						
	a	b	c	d	e	f	g
TN (mgL^{-1})	$2.46±0.27^{a}$	$1.91±0.09^{b}$	$1.33±0.24^{c}$	$1.06±0.07^{c}$	$0.67±0.03^{d}$	$0.54±0.04^{de}$	$0.34±0.02^{f}$
TP (mgL^{-1})	$0.17±0.01^{a}$	$0.16±0.01^{b}$	$0.15±0.01^{b}$	$0.14±0.01^{c}$	$0.09±0.01^{d}$	$0.08±0.01^{e}$	$0.06±0.01^{f}$
TDP (mgL^{-1})	$0.08±0.01^{a}$	$0.06±0.01^{b}$	$0.05±0.01^{c}$	$0.05±0.01^{cd}$	$0.04±0.01^{de}$	$0.04±0.01^{e}$	$0.02±0.01^{f}$
DRP(mgL^{-1})	$0.08±0.01^{a}$	$0.05±0.01^{b}$	$0.04±0.01^{bc}$	$0.04±0.01^{cd}$	$0.03±0.01^{de}$	$0.03±0.01^{e}$	$0.01±0.01^{f}$
NH_3^{+}-N(mgL^{-1})	$0.81±0.03^{a}$	$0.35±0.01^{b}$	$0.28±0.02^{c}$	$0.16±0.01^{d}$	$0.12±0.01^{e}$	$0.08±0.01^{f}$	$0.05±0.01^{f}$
NO_3^{-}(mgL^{-1})	$0.83±0.23^{a}$	$0.7±0.01^{ab}$	$0.6±0.01^{bc}$	$0.5±0.01^{c}$	$0.1±0.01^{d}$	$0.1±0.01^{d}$	$0.1±0.01^{d}$
COD (mgL^{-1})	$62.33±2.52^{a}$	$57.67±0.58^{ab}$	$56.67±0.58^{ab}$	$53.33±2.31^{c}$	$40.33±7.09^{d}$	$26±4.36^{e}$	$3.33±2.52^{f}$
DO (mgL^{-1})	$3.23±0.3^{bc}$	$2.94±0.25^{c}$	$0.19±0.01^{e}$	$3.67±0.16^{a}$	$3.55±0.02^{ab}$	$1.46±0.03^{d}$	$2.71±0.15^{c}$
pH	$8.72±0.03^{a}$	$8.29±0.16^{b}$	$8.65±0.03^{a}$	$8.11±0.03^{bc}$	$7.95±0.08^{cd}$	$7.5±0.17^{f}$	$7.77±0.09^{d}$
T (°C)	$9.85±0.01^{c}$	$9.74±0.02^{d}$	$10.24±0.03^{b}$	$9.13±0.02^{f}$	$10.46±0.02^{a}$	$9.56±0.01^{de}$	$9.45±0.08^{de}$
ORP (mV)	$201.77±1.3^{a}$	$199.8±1.35^{a}$	$-56.13±1.05^{f}$	$70.97±1.45^{e}$	$148±2.85^{d}$	$186.03±20.91^{ab}$	$178.97±1.86^{b}$
